# Knowledge and perception of Sepsis among Doctors in Karachi Pakistan

**DOI:** 10.12669/pjms.38.ICON-2022.5775

**Published:** 2022-01

**Authors:** Faiza Ahmed, Lubna Abbasi, Fivzia Herekar, Ahsun Jiwani, Muhammad Junaid Patel

**Affiliations:** 1 Dr. Faiza Ahmed, (FCPS II Trainee Year 4, Internal Medicine), Department of Internal Medicine, Indus Hospital and Health Network, Karachi, Pakistan; 2 Dr. Lubna Abbasi, FCPS (Internal Medicine/Rheumatology), Department of Internal Medicine, Indus Hospital and Health Network, Karachi, Pakistan; 3 Dr. Fivzia Herekar, FCPS (Internal Medicine/Infectious Disease), Department of Internal Medicine, Indus Hospital and Health Network, Karachi, Pakistan; 4 Mr. Ahsun Jiwani, MSc. Indus Hospital Research Center, Indus Hospital and Health Network, Karachi, Pakistan; 5 Dr. Muhammad Junaid Patel, (Diplomat American Board of Internal Medicine), Department of Internal Medicine, Indus Hospital and Health Network, Karachi, Pakistan

**Keywords:** Sepsis, Septic Shock, Critical Care, Intensivists, Physicians knowledge

## Abstract

**Objectives:**

To assess knowledge and perception among Pakistani physicians towards sepsis.

**Methods:**

This cross-sectional study was conducted in Indus Hospital and Health Networks from September 2020 to March 2021. The International Sepsis Survey questionnaire was adapted, and its link was sent to trainee physicians as well as specialists, and consultants practicing in various hospitals via social media. Knowledge and perception were scored and 50% was considered the cut-off score for adequacy. Data was analyzed using SPSS version 26.

**Results:**

Analysis was done on 222 respondents who completed the survey. 37.9% of the participants had adequate knowledge. Knowledge regarding sepsis was significantly associated with specialty, ICU/CCU/HDU, and work experience (P-value <0.0001). More recent trainee physicians and those with more experience in critical care areas demonstrated better knowledge. Over 2/3^rd^ of the respondents strongly agreed that sepsis remains one of the unmet needs in critical care today.

**Conclusion:**

A common belief exists that sepsis remains a challenge to treat among doctors. Moreover, there is consensus that it is the most frequently miss diagnosed condition in critical care and a dire need exists for its early diagnosis. Additionally, prompt management of presumed sepsis is imperative to improve outcomes.

## INTRODUCTION

Sepsis is considered one of the most life-threatening situations in critically ill patients. Being a medical emergency, delayed diagnosis and management are associated with higher mortality rates. Despite evidence-based management guidelines, sepsis remains a leading cause of death with mortality rate ranging between 22.8% to 48.7%.^1^-^6^ Identifying sepsis is challenging, given that its clinical presentation is variable and there is no gold standard for diagnosis. Additionally, the complexity and diversity of the disease further increases the difficulty for health care providers to diagnose it.^7^

Sepsis-related morbidity and mortality can be reduced through early treatment using protocols with well-established therapeutic targets. However, early intervention calls for prompt recognition by the team managing the patient. Studies have been conducted to assess the ability to recognize patients with sepsis and have suggested that knowledge of sepsis and its clinical forms of presentation is limited among health care professionals. A large Brazilian study showed a significant difference in knowledge existed among physicians with those working at university hospitals having better knowledge when compared to those in public hospitals.^8^

A recent study from Karachi concluded that half of the resident physicians included had excellent knowledge of the sepsis bundle.^9^ Another study conducted in Nepal showed that the healthcare workers who were or had previously worked in the critical areas such as emergency and ICUs, had better knowledge (31.7%) than those who were working in less critical or general areas (14.2%).^10^

There is a need for physicians from all specialties to recognize the early signs of sepsis, and make timely diagnosis and treatment possible for a better prognosis.^7^,^8^ A thorough understanding of its definitions and a more comprehensive perception about the disease itself is crucial to prepare our doctors for better management of sepsis. This research aims to establish the gaps in the knowledge and perception of healthcare professionals regarding sepsis and their ability to identify sepsis.

## METHODS

A cross-sectional survey was conducted between September 2020 to March 2021. The survey questions were adapted from the International Sepsis Survey^7^ after discussion with a senior internal medicine consultant. An electronic form was designed using REDCap software and the survey link was then sent to doctors working in different hospitals in Pakistan. Specifically, the survey was circulated via media links, such as email and other social media platforms like WhatsApp and Facebook to trainee physicians at the postgraduate level, specialist physicians and consultants of Internal Medicine, Critical care, Anesthesia, General Surgery, Orthopedics. Undergraduate medical students, nurses, paramedical staff, physiotherapists, and other non-healthcare-related personal were excluded from the study. Ethical approval was taken from our Institutional Review Board (IRD_IRB_2019_11_001)

Data was entered and analyzed by using SPSS statistical package version 26 software. Mean ± SD or median (IQR) was computed as appropriate for all the quantitative variables. Frequencies and percentages were calculated for all the categorical variables. The pre-coded questionnaire was adapted with questions regarding misattribution of sepsis symptoms to other conditions, definitions of sepsis, and knowledge of bacterial culture for diagnosis were used to assess knowledge (q17, 22, 23, 24, and 32.1) whereas the remaining questions of the Poeze et al’s survey questionnaire^7^ were used to assess perception and attitude of physicians regarding diagnosis and treatment of sepsis. Responses to the International Sepsis survey evaluating knowledge were based on SIRS, SOFA, and qsofa scoring systems.^11^ In the knowledge section, participants who correctly answered 50% of the questions were considered having adequate knowledge as 50% was the cutoff for positive or negative perception. Chi-Square test was applied as appropriate to detect the significant associations of covariates with knowledge and perception. P-value <0.05 was considered significant.

## RESULTS

In this survey, 355 doctors participated, and only 222 (62.52%) completed the study. The analysis is based on 222 participants. Over 58% of the respondents were women. Mean age was 30±4.2 years, with nearly 80% who responded being 30 years or younger. The mean duration of practice was 5 ±4 years. Nearly ¾ of those who responded were practicing in private institutes. Most of the respondents were residents (86.5%), with less than half being year-1 residents (42.3%). Half of the respondents (54.4%) had working experience in either ICU, CCU, or HDUs. (Table-I).

**Table I:** Demographic Characteristics of the Study Participants.

Variable	n (%)
**Age (in years)**	
20-30	179 (80.6%)
31-40	34 (15.3%)
More than 40	09 (4.1%)
**Gender**	
Female	128 (57.7%)
Male	94 (42.3%)
**Type of Institute**	
Private	159 (71.6%)
Public	63 (28.4%)
**Position**	
Consultant/ Specialist	30 (13.5%)
Resident	192 (86.5%)
**Residency Status**	
R1	94 (42.3%)
R2	56 (25.2%)
R3	30 (13.5%)
R4 and above	12 (6.3%)
**Specialty**	
Anesthesiology	34 (15.3%)
Emergency	23 (10.4%)
Family Medicine	21 (9.5%)
General Surgery	21 (9.5%)
Internal Medicine	67 (30.2%)
Pulmonologist	16 (7.3%)
Others	40 (18.02%)
**Medical practice duration**	
2 or less years	25 (11.3%)
3-5 years	150 (67.6%)
6 years or more	47 (21.2%)
**Working experience ICU/CCU/HDU**	121 (54.5%)
**Knowledge regarding sepsis**	
Adequate knowledge	84 (37.9%)
Inadequate knowledge	138 (62.2%)
**Perception regarding sepsis diagnosis & treatment**	
Negative perception	69 (31.1%)
Positive perception	153 (68.9%)

Overall, 38% had adequate knowledge regarding sepsis, and 69% had a positive perception regarding current sepsis diagnosis and treatment. A significant association was found between specialty (ICU/CCU/HDU), working experience, and knowledge, regarding sepsis (p<0.0001). Furthermore, compared to other age groups, a greater proportion of participants in the 31-40-year age group had inadequate knowledge related to sepsis compared to younger respondents. Moreover, the doctors working in the private settings had more knowledge (71%) as compared to the doctors of public hospitals. In terms of the departments, the Internal medicine department had the highest proportion of doctors with adequate knowledge (37%), followed by anesthesiology (21%) Table-II.

**Table II:** Association of participant characteristics with Sepsis related Knowledge and Perception.

Variables		Knowledge		P values	Perception		P values
	
		Inadequate n=138	Adequate n=84		Negative n=69	Positive n=153	
Age Groups	20-30	111 (80.4%)	68 (81%)	0.44^Ŧ^	58 (84.1%)	121 (79.1%)	0.39^Ŧ^
	31-40	23 (16.7%)	11 (13.1%)		10 (14.5%)	24 (15.7%)	
	> 40	04 (2.9%)	05 (06%)		01 (1.4%)	08 (5.2%)	
Gender	Female	82 (59.4%)	46 (54.8%)	0.49^Ŧ^	43 (62.3%)	85 (55.6%)	0.34^Ŧ^
	Male	56 (40.6%)	38 (45.2%)		26 (37.7%)	68 (44.4%)	
Type of Institution	Private	99 (71.7%)	60 (71.4%)	0.96^Ŧ^	54 (78.3%)	105 (68.6%)	0.14^Ŧ^
	Public	39 (28.3%)	24 (28.6%)		15 (21.7%)	48 (31.4%)	
Position	Consultant/Specialist	21 (15.2%)	09 (10.7%)	0.34^Ŧ^	08 (11.5%)	22 (14.4%)	0.57^Ŧ^
	Resident	117 (84.8%)	75 (89.3%)		61 (88.4%)	131 (85.6%)
Year of residency	R1	62 (44.9%)	32 (38.1%)	0.18^Ŧ^	28 (40.6%)	66 (43.1%)	0.83^Ŧ^
	R2	28 (20.3%)	28 (33.3%)		19 (27.5%)	37 (24.2%)	
	R3	18 (13%)	12 (14.3%)		11 (15.9%)	19 (12.4%)	
	R4 and above	09 (7.7%)	03 (4%)		03 (4.9%)	09 (6.8%)	
Specialty	Anesthesiology	16 (11.6%)	18 (21.4%)	<0.0001^Ŧ **^	12 (17.4%)	22 (14.4%)	0.23^Ŧ^
	Emergency	14 (10.1%)	09 (10.7%)		04 (5.8%)	19 (12.4%)	
	Family medicine	20 (14.5%)	01 (1.2%)		10 (14.5%)	11 (7.2%)	
	General surgery	12 (8.7%)	09 (10.7%)		06 (8.7%)	15 (9.8%)	
	Internal medicine	36 (26.1%)	31 (36.9%)		18 (26.1%)	49 (32%)	
	Pulmonologist	04 (2.9%)	12 (14.3%)		03 (4.3%)	13 (8.5%)	
	Others	36 (26.1%)	04 (4.7%)		16 (23.2%)	24 (15.7%)	
Practice duration	2 or less years	19 (13.8%)	06 (7.1%)	0.28^Ŧ^	07 (10.1%)	18 (11.8%)	0.93^Ŧ^
	3-5 years	92 (66.7%)	58 (69%)		47 (68.1%)	103 (67.3%)	
	6 years or more	27 (19.5%)	20 (23.8%)		15 (21.7%)	32 (20.9%)	
Working experience ICU/CCU/HDU		62 (44.9%)	59 (70.2%)	<0.0001^Ŧ**^	28(40.6%)	93 (60.8%)	0.005^Ŧ*^

Ŧ Chi-Square,

* p-value<0.05,

** p-value<0.0001

Regarding questions assessing knowledge, one fourth (¼) of the participants identified the infection as the leading cause of sepsis, followed by bacteremia/bacteria (20.7%) and immunocompromised state (13.5%). When asked about sepsis’s major signs and symptoms, more than half of the participants identified the three major symptoms correctly, i.e., fever (82%), tachycardia (54.5%), and hypotension (54%) (Table-III). When asked about misattributing the symptoms of sepsis to other conditions, only 32.9% of the participants strongly agreed to it (Table-IV).

**Table III:** Based upon everything you know about sepsis?

Responses	Frequency (%)
State of dysregulated host response to infection	140 (63.1%)
Infection leading to organ dysfunction/ failure	56 (25.2%)
Life threatening/ Critical condition that leads to potentially organ dysfunction caused by deregulated host response to infection	55 (24.8%)
Sepsis is a Systemic inflammatory response syndrome (SIRS)	49 (22.1%)
Multi-organ failure in response to bacteremia/ infection	42 (18.9%)
Severe Infection causing organ failure/ MODS/ SIRS/ circulating failure	25 (11.3%)
Clinical conditions (e.g. Vital instability, fever, increases TLC and SOFA score, abnormal heart and respiratory rate, metabolic collapse, poor immunity)	23 (10.4%)
Infection causing circulatory collapse	15 (6.8%)
Bacterial infection in blood	8 (3.6%)
**Causes of sepsis**	
Infection	57 (25.7%)
Bacteremia/Bacteria	46 (20.7%)
Immunocompromised state	30 (13.5%)
Micro organisms	25 (11.3%)
Low/poor immunity	23 (10.4%)
Pathogens	12 (5.4%)
Release of inflammatory markers	11 (5%)
Bacteria viruses	5 (2.3%)
Inflammation	2 (0.9%)
Other	20 (9%)
**Sign and Symptoms of sepsis**	
Fever	183 (82.4%)
Tachycardia	121 (54.5%)
Hypotension	120 (54.1%)
Tachypnea	56 (25.2%)
Altered mental status	23 (10.4%)
Respiratory distress	21 (9.5%)
Increased TLC	16 (7.2%)
Low GCS	10 (4.5%)
Unstable vitals	8 (3.6%)
Decrease urination/ AKI	9 (4.1%)
Organ/s failure	8 (3.6%)
Raised wbc count	5 (2.3%)
Low leukocyte count	3 (1.4%)
Shock	2 (0.9%)
Thrombocytopenia	2 (0.9%)
Any symptom of SIRS	2 (0.9%)
Lethargy	2 (0.9%)
**Which of the following therapies do you yourself use to treat these sepsis patients?**	
Antishock/organ support therapy	114 (51.4%)
Antibiotics	76 (34.2%)
Invasive surgical/radiological therapy	25 (11.3%)
Depend upon the patient	7 (3.2%)

**Table IV:** Sepsis related perception of study subjects.

Responses	Strongly agree	Somewhat agree	Somewhat disagree	Strongly disagree	Don’t know
Sepsis is a leading cause of mortality compared to other conditions	141 (63.5%)	75 (33.8%)	5 (2.3%)	0 (0%)	1 (0.5%)
Sepsis treatment is one of the unmet needs in critical care today	149 (67.1%)	64 (28.8%)	7 (3.2%)	1 (0.5%)	1 (0.5%)
Sepsis is a significant burden on the healthcare system in my country	172 (77.5%)	43 (19.4%)	2 (0.9%)	1 (0.5%)	4 (1.8%)
The symptoms of sepsis can be easily misattributed to other conditions	73 (32.9%)	84 (37.8%)	24 (10.8%)	38 (17.1%)	3 (1.4%)
Patients need better monitoring in order to catch sepsis at the earliest possible stage	187 (84.2%)	34 (15.3%)	1 (0.5%)	0 (0%)	0 (0%)
Patients are often being treated too late to reverse the onset of sepsis	159 (71.6%)	52 (23.4%)	9 (4.1%)	2 (0.9%)	0 (0%)
Families of sepsis patients find it difficult to understand sepsis	174 (78.4%)	42 (18.9%)	4 (1.8%)	2 (0.9%)	0 (0%)
The current treatment options for sepsis are not adequate.	24 (10.8%)	92 (41.4%)	88 (39.6%)	16 (7.2%)	2 (0.9%)
Doctors are eager for a breakthrough in treating sepsis?	151 (68%)	57 (25.7%)	12 (5.4%)	1 (0.5%)	1 (0.5%)
Sepsis is among the most challenging conditions a doctor can treat	151 (68%)	65 (29.3%)	6 (2.7%)	0 (0%)	0 (0%)

In response to questions assessing the study participants’ perceptions, 63% of the participants strongly believed that sepsis is a leading cause of mortality compared to other conditions. Furthermore, 72% of the doctors believed that patients are often being treated too late to reverse the onset of sepsis, and 84% of the participants agreed that patients need better monitoring to catch sepsis at the earliest possible stage (Table-IV).

## DISCUSSION

A significant association was found between specialties, ICU/CCU/HDU working experience, and knowledge regarding sepsis, with half of the respondents having adequate knowledge regarding the detection and management of sepsis. Participants working in fields with less interaction with a sepsis patient, such as Family Medicine, Radiology, Cardiology, Pediatric Medicine had inadequate knowledge compared to the other specialties like Anesthesia, Surgery, Internal Medicine, and Pulmonology. Similar results were reported by a Nepalese study where almost 46% of the participants who had worked in intensive care areas had adequate knowledge regarding sepsis.^10^

In our study, younger age group respondents had better knowledge than the older respondents. Our study also found that residents had more knowledge regarding sepsis than the consultants. Similar results were reported by a study conducted in Malaysia^12^ This could be because younger respondents were mainly residents who may have studied sepsis more recently, had more frequent encounters with septic patients due to their long hours of training, as well as differences in curriculum.

Almost 69% of the physicians in our study either strongly or somewhat believe that sepsis symptoms can easily be misattributed to other conditions. Similar results were reported by an international survey^7^ concluding that many disorders and syndromes mimic the presentation of sepsis.^13^ Thus making it difficult for physicians to address sepsis when it might be present.

Our study concluded that there is a lack of a standard definition of sepsis, and if a common definition is applied globally, it will help in the early detection and treatment of sepsis, with around three fourth (¾) of the participants agreeing to it. Other studies have found that although guidelines and definitions are in place, adherence to these guidelines is more of a concern and needs to be regularly audited.^14^

Bacterial culture was ranked as the most effective method for diagnosing sepsis by physicians. The second most effective method for diagnosing sepsis was hemodynamic monitoring, similar to previous study results.^7^,^12^ Although bacterial cultures are the most reliable method to diagnose infections, it hinders the early detection and treatment. Several studies have shown that early detection and treatment with antibiotics can reduce sepsis-related mortality.^14^ Therefore, other diagnostic modalities and strict monitoring of the early sign and symptoms should be incorporated more into practice for early detection of sepsis.^14^,^15^

Our study detected a statistically significant association between working experience of critical areas (ICU/CCU/HDU) with knowledge similar to other studies.^10^ The reason behind this phenomenon could be that the doctors working in critical areas get more exposure to patients with sepsis. They frequently get hands-on practice in detecting and managing patients suffering from sepsis compared to the doctors working in the less critical areas.

In light of the results of our study, we recommend training physicians in critical care areas more frequently to prepare them in detecting and managing sepsis. We would also suggest that the internationally accepted sepsis guidelines be implemented in the hospitals, and regular audits should be conducted to assess physicians’ compliance with those guidelines. Furthermore, conducting refresher courses on detection and management of sepsis should be done more often for trainees and consultants to improve their knowledge and familiarize them with the latest detection and treatment modalities.

### Limitation:

The major limitation of this study was that the sample analyzed was relatively small in terms of the target population, i.e., doctors working in hospitals. As it was an online survey, the response rate was on the lower side. Only 62% of the participants responded and filled the questionnaires sent to them. Secondly, our study did not assess the knowledge and perceptions of nurses who are an integral part of patient care management in the critical areas in our country. Moreover, our study did not assess what was being practiced by the study participants. Assessing practice is an integral part of a study when knowledge and perceptions are being assessed. It should be taken into account to identify the important data gaps to invest more time and resources in that component.

## CONCLUSION

Fundamental problems remain the same despite the gap of many years. Sepsis is yet one of the most frequently miss diagnosed condition in critical care, making the need for its early diagnosis imperative. Prompt management of presumed sepsis remains key to improving outcomes. Newer markers for the diagnosis of sepsis are not made readily available everywhere and hence not used as much. Had they been available, would they still have replaced the gold standard of blood culture? Probably not. Much needs to be done regarding early diagnosis, better management, and not to forget its prevention in individuals.

### Authors Contribution:

**FA:** Conceived, designed, collected data, and prepared the manuscript.

**FH:** Designed the questionnaire.

**AJ:** Analyzed and prepared the manuscript.

All authors reviewed and finalized approval of the manuscript.
